# Identification of Protein Kinase C Isoforms Involved in Type 1 Diabetic Encephalopathy in Mice

**DOI:** 10.1155/2018/8431249

**Published:** 2018-03-18

**Authors:** Jiayin Zheng, Yue Wang, Song Han, Yanlin Luo, Xiuli Sun, Ning Zhu, Li Zhao, Junfa Li

**Affiliations:** ^1^Department of Neurobiology and Center of Stroke, Beijing Institute for Brain Disorders, Capital Medical University, Beijing 100069, China; ^2^Department of Neurology, Beijing Chaoyang Hospital, Capital Medical University, Beijing, China

## Abstract

Diabetic encephalopathy is a complication of diabetes mellitus characterized by impaired cognitive functions. Protein kinase C (PKC) isoforms are rarely reported on diabetic encephalopathy, although they have been believed to play crucial roles in other diabetic complications. In this study, streptozotocin- (STZ-) induced diabetic mice were found to exhibit learning and memory deficits in the Morris water maze test. Meanwhile, the expression of cPKC*β*II, nPKC*ε*, and cPKC*γ* did not change in the hippocampus, cortex, and striatum at 2 and 8 weeks after STZ injection. The nPKC*ε* translocation to the membrane, where it is activated, was not altered in the above brain regions at 2 and 8 weeks after STZ injection. Nevertheless, cPKC*β*II translocation to the membrane was significantly decreased in the cortex and hippocampus at 8 weeks after STZ injection. The translocation of cPKC*γ* from the cytosol to the membrane was remarkably decreased in the hippocampus at 2 and 8 weeks and in the cortex and striatum at 8 weeks after STZ injection. In addition, deletion of cPKC*γ* aggravated the impairment of spatial learning and memory. In conclusion, our results suggest that the decrease in the activity of cPKC*β*II and cPKC*γ*, especially cPKC*γ*, may play key roles in the pathogenesis of diabetic encephalopathy.

## 1. Introduction

The prevalence rates of diabetes mellitus (DM) have increased rapidly over the past three decades in the world [1]. Some clinical studies have shown that high blood glucose increases the risk of Alzheimer's disease (AD) [2–4]. It has been manifested that type 1 diabetes mellitus (T1DM) has shown neurofibrillary and senile plaque, two major pathologic characteristics of AD, in the animal models [5, 6]. To describe cognitive impairment in diabetes, the term “diabetic encephalopathy” was introduced [7]. Now, diabetic encephalopathy is accepted as one of the most widespread diabetic complications in the central nervous system [8]. Because of a lack of effective treatment for diabetic encephalopathy, much interest now is focused on its molecular mechanism.

Constant hyperglycemia inside the cell increases the synthesis of diacylglycerol, which is one of the critical activating factors for protein kinase C (PKC) [9]. PKC, a family of Ser/Thr kinases that regulate a series of cellular processes, may play a key role in diabetes and its complications. According to the forms of activation, PKC isoforms are included in three groups: (1) conventional PKC isoforms (cPKC*α*, *β*I, *β*II, and *γ*) are activated by diacylglycerol, phosphatidylserine, and Ca^2+^ influx; (2) novel PKC isoforms (nPKC*δ*, *ε*, *η*, and *θ*) require diacylglycerol; and (3) atypical PKC isoforms (aPKC*ζ* and *ι*/*λ*) do not bind either second messengers. It has been demonstrated that the cPKC*β*II, cPKC*γ*, and nPKC*ε*, widely expressed in the nervous system, participate in many diabetic complications [10–14].

cPKC*β*II is the most studied isoform for diabetic complications. Deletion or inhibition of the cPKC*β*II can reduce glomerular, albuminuria, and mesangial expansion, which prevent the thickening of the glomerular basement membrane and the obliteration of glomerular capillaries in a diabetic kidney [15, 16]. The cPKC*β*II inhibitor can reverse acute blood glucose fluctuation-induced endothelial cell apoptosis increase, inflammatory cytokine level increase, and insulin signaling impairment, indicating that PKC*β*II may serve as a target for anticardiovascular diseases [11]. The cPKC*β*II inhibitor can also reduce the incidence of visual loss and prevent blood-retinal barrier breakdown in diabetic patients and animal models [17].

nPKC*ε* is highly relative to insulin resistance. The key role of nPKC*ε* for fat-induced hepatic insulin resistance is demonstrated with antisense oligonucleotide-mediated knockdown of nPKC*ε* in high-fat feeding rats [18]. Several studies in type 2 diabetic patients have demonstrated that activation of nPKC*ε* by hepatic accumulation of the diacylglycerol can result in hepatic insulin resistance and nonalcoholic fatty liver disease [19]. nPKC*ε* deletion also enhances the amplifying pathways of glucose-stimulated insulin secretion and lipolysis in diabetic mouse beta-cells [20].

Compared to other PKC isoforms, cPKC*γ* is located only in the neurons of the central nervous system. Some studies have reported that the inhibitors of cPKC*γ* could reverse the DM-associated chronic impairment of neurovascular coupling [10]. The increase in cPKC*γ* expression in a trigeminal spinal nucleus is associated with orofacial thermal hyperalgesia in diabetic mice [21]. The increase in cPKC*γ* activity is also associated with diabetic embryonic dysmorphogenesis in rat [22]. However, few studies investigate the roles of the three specific PKC isoforms in the diabetic encephalopathy. In this study, we explored whether cPKC*β*II, cPKC*γ*, and nPKC*ε* participated in the pathogenesis of type 1 diabetic encephalopathy induced by streptozocin (STZ) injection in mice.

## 2. Materials and Methods

### 2.1. Animals

C57BL/6 wild-type and cPKC*γ* knockout mice (male, 18–22 g) were purchased from Jackson Laboratory (Bar Harbor, Maine 04609, USA). Mice were housed, three per cage. The mice with free access to water and food were maintained in the Experimental Animal Center of Capital Medical University, the People's Republic of China. The experimental procedures were approved by the experimental animal ethics committee of Capital Medical University (permit number AEEI-2014-035) and conducted according to the recommendations in the Guide for the Care and Use of Laboratory Animals of the National Institutes of Health.

### 2.2. Induction of Type 1 Diabetes Mellitus in Mice

Animals were randomly divided into three groups, namely, the CON group (with citrate buffer treatment), T1DM group (with STZ treatment), and T1DM + cPKC*γ* group (cPKC*γ* knockout mice with STZ treatment). Following an overnight fast, T1DM mice were induced by low doses of freshly prepared STZ (50 mg/kg/day, i.p.), dissolved in a 0.1 M citrate buffer (pH 4.5), over five consecutive days. The control mice were only injected with citrate buffer (5 mL/kg/day, i.p.). The fasting blood glucose level was measured every week using a blood sample from the tail vein with the aid of a OneTouch® Ultra blood glucose meter (Milpitas, CA, USA). Mice with blood glucose levels over 11.1 mmol/L were considered diabetic and used for the study [23]. At 2 weeks and 8 weeks after STZ injection, the hippocampus, prefrontal cortex, and striatum of the mice in the CON and T1DM groups were prepared for the determination of PKC isoform expression levels and membrane translocation of PKC isoforms (*n* = 6 per group, resp.). At 8 weeks after STZ injection, the mice in the CON, T1DM, and T1DM + cPKC*γ* groups were tested in the Morris water maze (*n* = 12 per group, resp.).

### 2.3. Cytosolic and Membrane Fractions and Whole Tissue Homogenate Preparation

As previously reported [24], the dissected samples were first homogenized at 4°C in buffer A [50 mM Tris-Cl, pH 7.5, containing 1 mM DTT, 2 mM EDTA, 2 mM EGTA, 50 mM potassium fluoride (KF), 5 mM sodium pyrophosphate, and a protease inhibitor mixture]. Homogenates were centrifuged at 30,000*g* for 30 min, and the supernatant was collected as cytosolic fractions. The pellet fractions (membrane fractions) were resuspended in buffer A containing 2% SDS before being sonicated and centrifuged again. These two fractions were analyzed for membrane translocation of PKC isoforms. To analyze PKC isoform expression, the whole tissue was homogenized and sonicated in RIPA buffer. The concentration of protein was analyzed as previously reported [25].

### 2.4. Western Blot

As previously reported [26], the samples (30 *μ*g) were subjected to 10% SDS-PAGE and transferred onto polyvinylidene difluoride membrane (GE Healthcare, UK); the membrane was blocked with 10% nonfat milk for 1 h and probed with anti-cPKC*β*II (number sc-210, Santa Cruz Biotechnology, Santa Cruz, USA), cPKC*γ* (number sc-211, Santa Cruz Biotechnology, Santa Cruz, USA), and nPKC*ε* (number sc-214, Santa Cruz Biotechnology, Santa Cruz, USA). The horseradish peroxidase-conjugated goat anti-rabbit (number 31460, ThermoFisher Scientific, Rockford, USA) and anti-mouse IgG (number 31430, ThermoFisher Scientific, Rockford, USA) were used as second antibodies at a 1 : 4000 dilution for 1 h at room temperature. Signals were visualized via an enhanced chemiluminescent reagent solution (chemiluminescent HRP substrate, Millipore Corporation, USA). To verify the equal loading of protein, the blots were reprobed with primary monoclonal antibodies against *β*-actin (number 60008-1-lg, Proteintech, Rosemont, USA) or Na^+^/K^+^ ATPase (number 14418-1-AP, Proteintech, Rosemont, USA). The protein expression levels of PKC isoforms were calculated as 100% in the control group, and then the other groups were expressed as the percentage of the control group. Quantitative analysis for immunoblotting was done by Fusion Capt 16.15 software (Fusion FX6 XT, Vilber Lourmat, France).

### 2.5. Morris Water Maze

**Figure 1:**
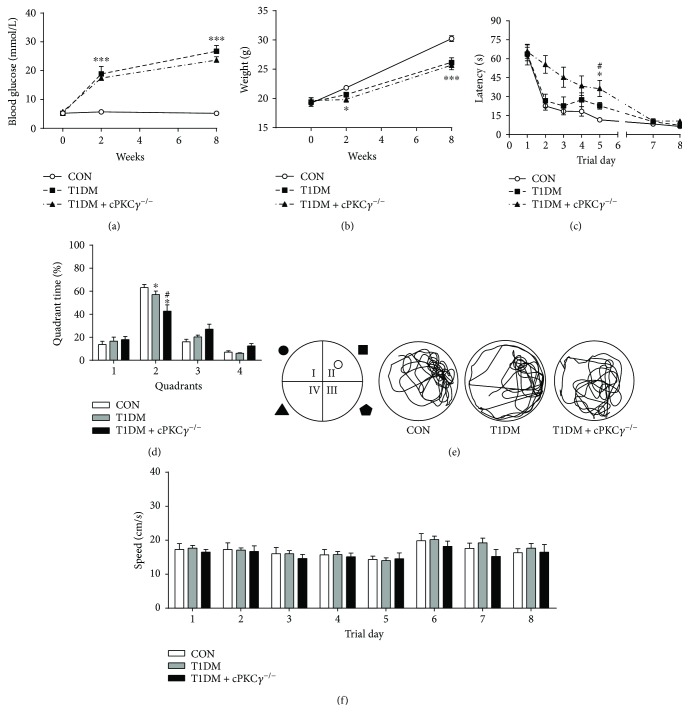
Changes of body weight, blood glucose, and spatial learning and memory deficits induced by STZ in mice. (a) Statistical results of blood glucose of each group. (b) Statistical results of body weight of each group. (c) The graph showed the escape latency of each group during 5 consecutive days of learning process and visible test (days 7 and 8). (d) Statistical results of the time spent in the target quadrant measured on day 6 by removing the platform. (e) The graph showed the swim path of each group on day 6. (f) Statistical results of speeds of each group every day. ^∗^*p* < 0.05 compared with the CON group, ^∗∗∗^*p* < 0.001 compared with the CON group, and ^#^*p* < 0.05 compared with the T1DM group. *n* = 12 per group.

At 8 weeks after STZ injection, spatial learning and memory were tested by the Morris water maze. Briefly, a custom-built water tank (122 cm diameter, 50 cm height) with a white nonreflective interior surface was filled with opaque water (19–22°C) containing nontoxic titanium white-colored dye. The experiment was performed in a room with low-light indirect lighting. Four different extramaze cues were fixed at four quartiles of the tank periphery (Figure 1(e)). In the target quadrant (quadrant II), a platform was submerged 1 cm below the water surface. The mice were trained to find the hidden platform for 5 consecutive days, four trials of 90 seconds per day. In each trial, the mouse started from one of the four quadrants facing the wall of the pool and ended when the mouse climbed on the platform. If the mice did not find the platform in 90 s, they were guided to the platform. To test reference memory, the platform was removed on day 6 and the percentage of time that the mice spent in each quadrant was recorded. The visible platform tests (with the platform marked) were given on day 7 and day 8 to evaluate sensorimotor abilities and motivation of the animals. Swim paths were recorded on a CCD camera and analyzed using WaterMaze 3 Software. Average swimming speed was determined to exclude motor impairments.

### 2.6. Statistics Analysis

The values were presented as the mean ± SEM. Statistical analysis of Western blot was conducted by a one-way analysis of variance followed by all pairwise multiple comparison procedures using the Bonferroni test. The statistical analysis of the Morris water maze was conducted by a two-way analysis of variance followed by all pairwise multiple comparison procedures using the Bonferroni test. All results were regarded as at least *p* < 0.05 was considered statistically significant.

## 3. Results

### 3.1. Biochemical Characteristics of the Mice

At the beginning of the experiment, there was no significant difference among the groups in blood glucose and body weight. Following STZ injection, the mice developed DM rapidly with a mean blood glucose of 13.13 ± 4.74 mmol/L on day 3 after STZ injection, 18.87 ± 3.79 mmol/L at 2 weeks, and 26.7 ± 5.70 mmol/L at 8 weeks (Figure 1(a)). As shown in Figure 1(b), the body weight of the T1DM group increased (from 19.38 ± 2.03 g to 26.12 ± 2.36 g at 8 weeks after STZ injection) but much more slowly than the increase of the CON group (from 19.18 ± 1.54 g to 30.22 ± 1.43 g at 8 weeks after STZ injection). There was no significant difference in blood glucose and body weight between the cPKC*γ*^+/+^ diabetic mice and the scPKC*γ*^−/−^ diabetic mice (Figures 1(a) and 1(b)).

### 3.2. Expression of PKC Isoforms in the Hippocampus, Prefrontal Cortex, and Striatum of the T1DM Mice

**Figure 2:**
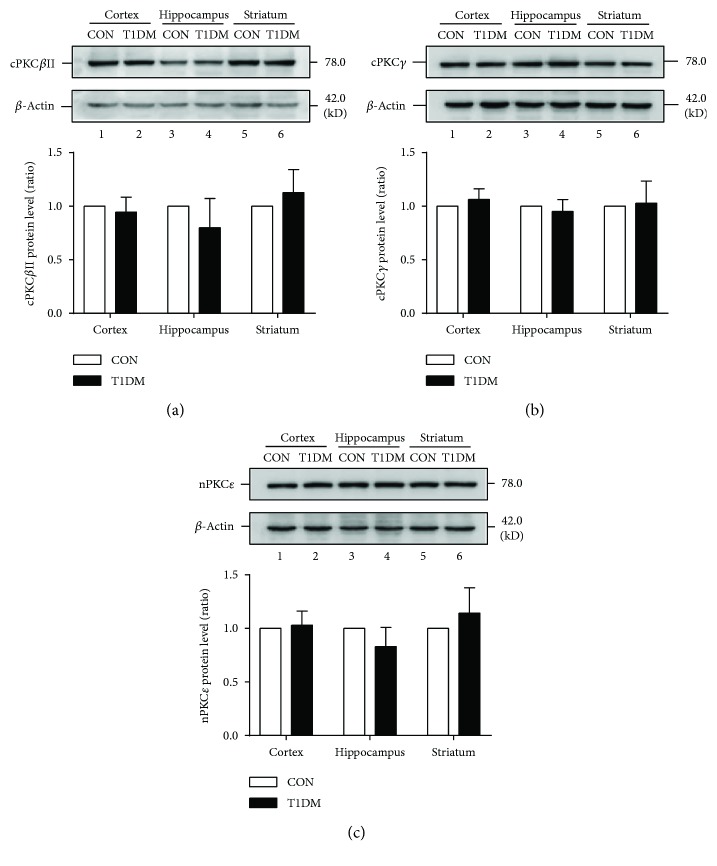
Expression of PKC isoforms in the cortex, hippocampus, and striatum at 2 weeks after STZ injection. (a) Representative and statistical results of Western blot analysis showed the levels of cPKC*β*II in the cortex, hippocampus, and striatum at 2 weeks after STZ injection. (b) Representative and statistical results of Western blot analysis showed the levels of cPKC*γ* in the cortex, hippocampus, and striatum at 2 weeks after STZ injection. (c) Representative and statistical results of Western blot analysis showed the levels of nPKC*ε* in the cortex, hippocampus, and striatum at 2 weeks after STZ injection. *n* = 6 per group.

**Figure 3:**
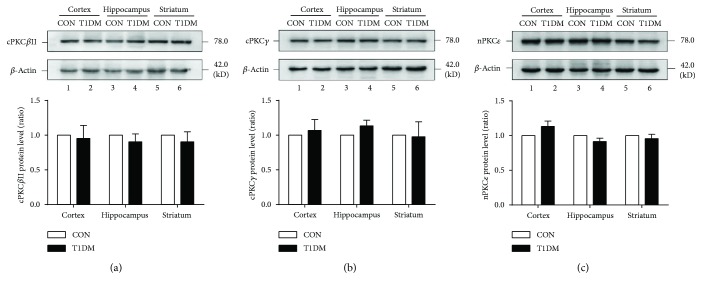
Expression of PKC isoforms in the cortex, hippocampus, and striatum at 8 weeks after STZ injection. (a) Representative and statistical results of Western blot analysis showed the levels of cPKC*β*II in the cortex, hippocampus, and striatum at 8 weeks after STZ injection. (b) Representative and statistical results of Western blot analysis showed the levels of cPKC*γ* in the cortex, hippocampus, and striatum at 8 weeks after STZ injection. (c) Representative and statistical results of Western blot analysis showed the levels of nPKC*ε* in the cortex, hippocampus, and striatum at 8 weeks after STZ injection. *n* = 6 per group.

In the next set of experiments, we observed whether diabetes mellitus affected the expression patterns of PKC isoforms in the hippocampus, prefrontal cortex, and striatum. As shown in Figures 2 and 3, we observed that the expression of cPKC*β*II, nPKC*ε*, and cPKC*γ* did not change in the above brain regions at 2 or 8 weeks after STZ injection. These results indicated that chronic T1DM did not affect the expression of the three PKC isoforms in the hippocampus, prefrontal cortex, and striatum.

### 3.3. Membrane Translocation of PKC Isoforms in the Hippocampus, Prefrontal Cortex, and Striatum of the T1DM Mice

The activation of PKC relates to a transition from the cytosolic autoinhibited form to the membrane-associated active form [27]. To figure out whether PKC isoforms are involved in type 1 diabetic encephalopathy, we observed the membrane translocation of PKC isoforms in the hippocampus, prefrontal cortex, and striatum of the T1DM mice.

**Figure 4:**
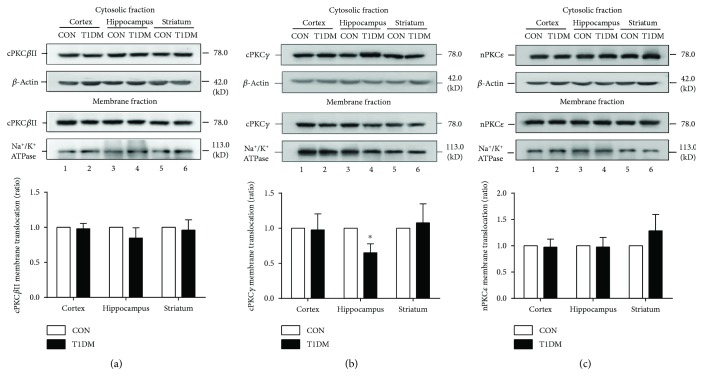
Membrane translocation of PKC isoforms in the cortex, hippocampus, and striatum at 2 weeks after STZ injection. (a) Representative and statistical results of Western blot analysis showed the membrane translocation of cPKC*β*II in the cortex, hippocampus, and striatum at 2 weeks after STZ injection. (b) Representative and statistical results of Western blot analysis showed the membrane translocation of cPKC*γ* in the cortex, hippocampus, and striatum at 2 weeks after STZ injection. (c) Representative and statistical results of Western blot analysis showed the membrane translocation of nPKC*ε* in the cortex, hippocampus, and striatum at 2 weeks after STZ injection. ^∗^*p* < 0.05 compared with the CON group. *n* = 6 per group.

**Figure 5:**
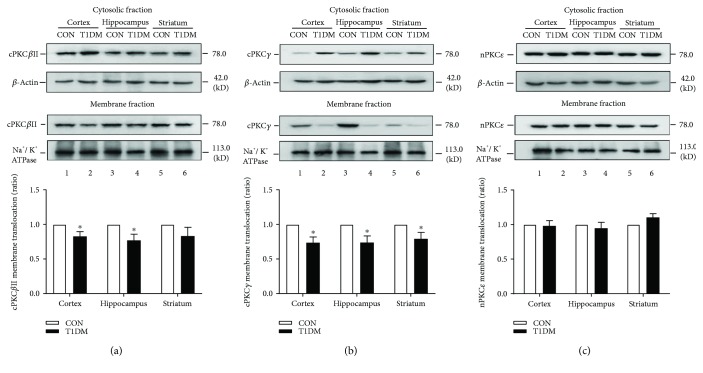
Membrane translocation of PKC isoforms in the cortex, hippocampus, and striatum at 8 weeks after STZ injection. (a) Representative and statistical results of Western blot analysis showed the membrane translocation of cPKC*β*II in the cortex, hippocampus, and striatum at 8 weeks after STZ injection. (b) Representative and statistical results of Western blot analysis showed the membrane translocation of cPKC*γ* in the cortex, hippocampus, and striatum at 8 weeks after STZ injection. (c) Representative and statistical results of Western blot analysis showed the membrane translocation of nPKC*ε* in the cortex, hippocampus, and striatum at 8 weeks after STZ injection. ^∗^*p* < 0.05 compared with the CON group. *n* = 6 per group.

As shown in Figures 4(c) and 5(c), chronic T1DM did not affect the membrane translocation of nPKC*ε* in the hippocampus, prefrontal cortex, and striatum at 2 weeks and 8 weeks after STZ injection. The ratio of membranal cPKC*β*II to cytosolic cPKC*β*II was not significantly altered in the three brain regions at 2 weeks (Figure 4(a)) but dramatically decreased in the prefrontal cortex and hippocampus at 8 weeks (Figure 5(a)). Moreover, chronic T1DM significantly reduced the ratio of membranal cPKC*γ* to cytosolic cPKC*γ* in the hippocampus at 2 weeks (Figure 4(b)) and in the prefrontal cortex, hippocampus, and striatum at 8 weeks (Figure 5(b)).

### 3.4. Effects of cPKC*γ* on Spatial Learning and Memory of Mice

To explore the role of cPKC*γ* in learning and memory, the Morris water maze test was used at 8 weeks after STZ injection. On day 5, the diabetic mice had a significantly increased latency to find the hidden platform compared to control mice (Figure 1(c)). Furthermore, the cPKC*γ*-deficient diabetic mice took more time to find the hidden platform than the wild-type diabetic mice (Figure 1(c)).

On day 6, swim patterns indicated that control mice swam immediately towards the target area, where the platform used was placed, tried to find the platform, and swam back and forth. In contrast, diabetic mice swam in random circles throughout the pool (Figure 1(e)). In addition, the cPKC*γ*^−/−^ diabetic mice performed worse than the wild-type diabetic mice (Figure 1(e)).

The percentage of the time spent swimming in the target quadrant (quadrant II) was significantly lower for diabetic mice than for control mice (Figure 1(d)). Moreover, cPKC*γ*^−/−^ diabetic mice spent less time in the target quadrant than cPKC*γ*^+/+^ diabetic mice (Figure 1(d)), again indicating memory impairment in the cPKC*γ* knockout mice.

As both the vision and sensorimotor abilities of mice could affect the reliability of Morris water maze tests, the visible platform tests (with the platform marked) were given on day 7 and day 8. There were no significant differences in escape latency among groups (Figure 1(c)). As shown in Figure 1(f), no significant differences in average speed were observed among groups in 8 days. These results suggested that chronic T1DM could induce spatial learning and memory deficits of mice without affecting their visual and sensorimotor abilities and cPKC*γ* deficiency aggravated the spatial learning and memory impairment of T1DM mice.

## 4. Discussion

Several studies have reported that the expression of cPKC*β*II, cPKC*γ*, and nPKC*ε* is altered by hyperglycemia. The expression of cPKC*β*II increases in the renal tissues of diabetic rats on 21 days after STZ injection [28]. The nPKC*ε* level increases in the skeletal muscle of nutritionally induced diabetic rats in 3 weeks [29] and vessels of spontaneously diabetic rats [30]. And the expression of cPKC*γ* is also found to increase in the trigeminal spinal nucleus of T1DM mice on 14 days after STZ injection [21]. Interestingly, the expression of cPKC*β*II, cPKC*γ*, and nPKC*ε* may not change in the cerebral cortex, but the levels of cPKC*γ* and nPKC*ε* are decreased in the glio-pial tissue of T1DM rats at 16 weeks after STZ injection [31]. In this study, we explored the expression and activation of cPKC*β*II, cPKC*γ*, and nPKC*ε* in the hippocampus, prefrontal cortex, and striatum, the regions important in the cognitive processes of learning, memory, and decision-making [32–34]. We observed that T1DM did not significantly change the protein expression of cPKC*β*II, cPKC*γ*, and nPKC*ε* in the three brain regions at 2 weeks after STZ injection. Next, we prolonged the observation time. But we still did not find significant changes in the protein expression of cPKC*β*II, cPKC*γ*, and nPKC*ε* at 8 weeks after STZ injection.

Activation of PKC is associated with the translocation of enzymes from the cytosol to the membrane. The membrane translocation or activation of PKC isoforms is linked to the development of pathologies in diabetes. The acute blood glucose fluctuation significantly increased cPKC*β*II membrane translocation, which causes the apoptosis of vascular endothelial cell through increasing oxidative stress and induces impairment of insulin signaling in diabetic rats [11]. Activation of cPKC*β*II by hyperglycemia upregulates caspase 8-induced apoptosis in blood-brain barrier endothelial cells [35]. Activation of nPKC*ε*, as a result of the accumulation of diacylglycerol, results in hepatic insulin resistance and nonalcoholic fatty liver disease in patients and type 2 diabetic mice [19, 36]. Activation of cPKC*γ* by early STZ-induced diabetes causes the inhibition of lens gap junction activity in rats [37]. In this study, we found that T1DM mice exhibited spatial learning and memory impairment in the Morris water maze at 8 weeks after STZ injection. Meanwhile, T1DM failed to affect nPKC*ε* membrane translocation in the hippocampus, prefrontal cortex, and striatum at 8 weeks. However, the translocation of cPKC*β*II and cPKC*γ* from the cytosol to the membrane was significantly decreased in the brain of T1DM mice at 8 weeks, indicating that they might be involved in type 1 diabetic encephalopathy.

It is well accepted that cPKC*β*II is essential for learning and memory formation [38, 39]. The downregulation of cPKC*β*II in the hippocampus of offspring, especially in the CA3, CA1, and dentate gyrus, the regions important in the entorhinal-hippocampal trisynaptic circuit, is responsible for impaired learning and memory of prenatally stressed offspring [40]. The levels of cPKC*β*II decrease in the striatum and cortex of transgenic Huntington's disease mice, which is associated with impaired information storage and memory disorder [41]. The baseline asymmetry and lateralized changes of cPKC*β*II in the rat amygdale are associated with the cue and context in a classical fear conditioning paradigm [42]. And furthermore, the cPKC*β*II knockout mice also exhibit a loss of learning and suffer deficits in both cued and contextual fear conditioning [43].

cPKC*γ* is a neuron-specific isoform, and its activity is suggested to be related to spatial memory in aged rats [44, 45]. Activation of cPKC*γ* in the CA1 region participates in postsynaptic plasticity associated with spatial experiences and learning [46]. Young rats, those with the best spatial memory, are those with the highest concentrations of cPKC*γ* in the particulate fraction. Conversely, aged rats with poor spatial memory increase hippocampal cPKC*γ* concentrations in soluble fraction in comparison with young rats, which indicates that the decrease in cPKC*γ* activity is correlated with memory impairment [47]. In addition, cPKC*γ* is found to participate in the reversal of learning and memory deficits by intranasal insulin treatment in AD mice [48]. In this study, we found that the decrease in cPKC*γ* activity induced by T1DM came earlier and occurred in more brain regions than cPKC*β*II, suggesting that cPKC*γ* played a bigger role in the diabetic encephalopathy. Therefore, we deleted the cPKC*γ* gene in mice and then found that the spatial learning and memory of cPKC*γ*^−/−^ diabetic mice are worse than those of wild diabetic mice.

Elevated glucose level and impaired glucose tolerance can affect the spatial learning and memory, which is associated with hippocampal apoptosis [49–52]. But in this study, we found that cPKC*γ* did not affect blood glucose level, demonstrating that the effect of cPKC*γ* on cognitive function is irrelevant to blood glucose. PKC activators have been reported to improve cognitive function by restoring PKC signaling and downstream activity, including stimulation of synaptic plasticity and development [53], reduction in accumulation of neurotoxic amyloid *β*, regulation of tau protein hyperphosphorylation [38], and support of antiapoptotic processes [54]. Further studies about the mechanism how cPKC*γ* can enhance spatial memory performance in T1DM mice are required.

## 5. Conclusion

Our results indicate that the activation of cPKC*β*II and cPKC*γ*, not nPKC*ε*, may be involved in the development of the diabetic encephalopathy in mice. cPKC*γ* can dramatically improve learning and memory performances impaired by diabetes mellitus in mice.
